# Preclinical rationale for entinostat in embryonal rhabdomyosarcoma

**DOI:** 10.1186/s13395-019-0198-x

**Published:** 2019-05-21

**Authors:** Narendra Bharathy, Noah E. Berlow, Eric Wang, Jinu Abraham, Teagan P. Settelmeyer, Jody E. Hooper, Matthew N. Svalina, Zia Bajwa, Martin W. Goros, Brian S. Hernandez, Johannes E. Wolff, Ranadip Pal, Angela M. Davies, Arya Ashok, Darnell Bushby, Maria Mancini, Christopher Noakes, Neal C. Goodwin, Peter Ordentlich, James Keck, Douglas S. Hawkins, Erin R. Rudzinski, Atiya Mansoor, Theodore J. Perkins, Christopher R. Vakoc, Joel E. Michalek, Charles Keller

**Affiliations:** 1 Children’s Cancer Therapy Development Institute, 12655 Sw Beaverdam Rd. W, Beaverton, OR 97005 USA; 2 Cold Spring Harbor Laboratory, Cold Spring Harbor, NY 11724 USA; 3 Department of Pediatrics, Oregon Health & Science University, Portland, OR 97239 USA; 4 Department of Pathology, Johns Hopkins University School of Medicine, Baltimore, MD 21287 USA; 5 Department of Pathology, Oregon Health & Science University, Portland, OR 97239 USA; 6 Department of Epidemiology and Biostatistics, University of Texas Health Science Center, San Antonio, TX 78229 USA; 7 Department of Pediatric Hematology Oncology and Blood and Marrow Transplantation, Cleveland Clinic Children’s, Cleveland, OH 44195 USA; 8 Present Address: AbbVie, North Chicago, IL 60064 USA; 9 Electrical and Computer Engineering, Texas Tech University, Lubbock, TX 79409 USA; 10 Champions Oncology, Rockville, MD 20850 USA; 11 Syndax Pharmaceuticals, Waltham, MA 02451 USA; 12 The Jackson Laboratory, Sacramento, CA 95838 USA; 13 Seattle Children’s Hospital, Seattle, WA 98105 USA; 14 Regenerative Medicine Program, Ottawa Hospital Research Institute, Ottawa, K1H 8L6 Canada; 15 Department of Biochemistry, Microbiology and Immunology, Faculty of Medicine, University of Ottawa, Ottawa, K1H 8M5 Canada

**Keywords:** Embryonal rhabdomyosarcoma (eRMS), Entinostat, Vincristine, HDAC3, Patient-derived xenograft (PDX)

## Abstract

**Background:**

Rhabdomyosarcoma (RMS) is the most common soft tissue sarcoma in the pediatric cancer population. Survival among metastatic RMS patients has remained dismal yet unimproved for years. We previously identified the class I-specific histone deacetylase inhibitor, entinostat (ENT), as a pharmacological agent that transcriptionally suppresses the *PAX3:FOXO1* tumor-initiating fusion gene found in alveolar rhabdomyosarcoma (aRMS), and we further investigated the mechanism by which ENT suppresses *PAX3:FOXO1* oncogene and demonstrated the preclinical efficacy of ENT in RMS orthotopic allograft and patient-derived xenograft (PDX) models. In this study, we investigated whether ENT also has antitumor activity in fusion-negative eRMS orthotopic allografts and PDX models either as a single agent or in combination with vincristine (VCR).

**Methods:**

We tested the efficacy of ENT and VCR as single agents and in combination in orthotopic allograft and PDX mouse models of eRMS. We then performed CRISPR screening to identify which HDAC among the class I HDACs is responsible for tumor growth inhibition in eRMS. To analyze whether ENT treatment as a single agent or in combination with VCR induces myogenic differentiation, we performed hematoxylin and eosin (H&E) staining in tumors.

**Results:**

ENT in combination with the chemotherapy VCR has synergistic antitumor activity in a subset of fusion-negative eRMS in orthotopic “allografts,” although PDX mouse models were too hypersensitive to the VCR dose used to detect synergy. Mechanistic studies involving CRISPR suggest that HDAC3 inhibition is the primary mechanism of cell-autonomous cytoreduction in eRMS. Following cytoreduction in vivo, residual tumor cells in the allograft models treated with chemotherapy undergo a dramatic, entinostat-induced (70–100%) conversion to non-proliferative rhabdomyoblasts.

**Conclusion:**

Our results suggest that the targeting class I HDACs may provide a therapeutic benefit for selected patients with eRMS. ENT’s preclinical in vivo efficacy makes ENT a rational drug candidate in a phase II clinical trial for eRMS.

**Electronic supplementary material:**

The online version of this article (10.1186/s13395-019-0198-x) contains supplementary material, which is available to authorized users.

## Background

Rhabdomyosarcoma (RMS) is among the most common causes of death in pediatric population with cancer. RMS is comprised of two main histological subtypes, embryonal RMS (eRMS) and alveolar RMS (aRMS) with eRMS comprising half of all RMS cases [1]. The cells of eRMS are reminiscent of myogenic precursors delaminating from the lateral dermomyotome of a human 6- to 8-week-old embryo [2]. eRMS exhibits 11p15 loss of heterozygosity [3, 4] and/or mutations in the components of RAS pathway including *NRAS*, *KRAS*, *HRAS*, *FGFR4*, *PIK3CA*, *CTNNB1*, *FBXW7*, and *BCOR* [5, 6]. Metastatic RMS patients have the poorest prognosis, with no improvements in outcome in 46 years [7–9]. The long-term survival rate for metastatic eRMS is 40% [7, 9–12].

With the addition of a targeted therapy, short-term survival in relapsed RMS is improving measurably; notably, the ARST0921 Children’s Oncology Group (COG) clinical trial for relapsed RMS was stopped early. The 6-month event-free survival (EFS) for temsirolimus plus vinorelbine and cyclophosphamide chemotherapy was superior to the 6-month EFS for bevacizumab plus the same chemotherapy (65% vs 50%, two-sided *p* value = 0.0031) [13]. The next task is to determine whether these stepwise changes in 6-month EFS will result in improvements over chemotherapy-only long-term survival rates for metastatic aRMS and eRMS [1, 3]. Nevertheless, other new agents are also needed.

Our recent study established the antitumor efficacy of class I-specific histone deacetylase (HDAC) inhibitor, entinostat (ENT) with the chemotherapy, vincristine (VCR) in preclinical cell and mouse models and found HDAC3 inhibition as the primary mechanism of decreasing PAX3:FOXO1 expression in fusion positive aRMS [14]. Herein, we demonstrate that ENT in combination with the chemotherapy VCR has synergistic antitumor activity in a orthotopic fusion-negative eRMS mouse model although our studies of patient-derived xenograft (PDX) mouse models had too strong of VCR single-agent activity to detect synergy. Mechanistic studies involving CRISPR suggest that HDAC3 inhibition is the primary mechanism of cell-autonomous cytoreduction in eRMS. Our findings demonstrate that targeting class I HDACs using ENT is a novel, clinically feasible epigenetic therapy for children with fusion-negative eRMS—a result which is in line with our parallel studies in fusion-positive RMS [14].

## Methods

### Cell culture

Murine primary tumor cell cultures U57810 and U37125 were generated as described previously [15]. Briefly, for the establishment of murine eRMS primary cell cultures, tumor samples were minced into small fragments followed by collagenase treatment (0.5%) overnight at 4 °C. The disassociated cells were incubated in DMEM supplemented with 10% fetal bovine serum (FBS) and penicillin (100 U/mL)/streptomycin (100 μg/mL) in 5% CO_2_ in air at 37 °C. Human eRMS RD and Rh18 cell lines were cultured in RPMI 1640 growth medium supplemented with 10% FBS and 1% penicillin/streptomycin and incubated at 37 °C and 5% CO_2_. Primary Human Skeletal Muscle Myoblasts (HSMM) were cultured in growth medium (Cell Applications) supplemented with 10% FBS and 1% penicillin/streptomycin and incubated at 37 °C and 5% CO_2_.

### ENT treatment

HDAC inhibitor (HDACi): ENT (MS-275) was obtained from Selleckchem (S1053).

### RNA extraction and RT-PCR

For murine eRMS cell treatment studies, U57810 cells were treated with VCR (2.5 nM), ENT (400 nM), or VCR and ENT (2.5 nM and 400 nM, respectively) for 6 days. RT-PCR was performed for *Myoglobin*, *Myh1*, *MyoD*, and *Myogenin* relative to *Gapdh* using probes from SYBR green (Thermo Fisher Scientific).

### VCR: chemotherapy agent

VCR was obtained from Sigma (V8879).

### siRNA-mediated knockdown of HDAC3

For silencing HDAC3, Rh30, RD, and HSMM cells were transfected with 100 nM HDAC3 (L-003496-00-0005, 5 nmol) SMART pool siRNA reagent (a pool of four siRNA duplexes all designed to target distinct sites within the specific gene of interest) (Dharmacon) versus scrambled siRNA (Dharmacon) using Lipofectamine RNAimax (Invitrogen). siRNA lysates were subjected to Western blotting using anti-HDAC3 antibody (Santa Cruz Biotechnology).

### Orthotopic allograft studies

Allograft studies were conducted with IACUC approval at the Oregon Health & Science University. The orthotopic allograft mouse model of eRMS (U57810, genotype *Myf5Cre, p53*) was generated by injecting SCID Hairless Outbred (SHO) mice at 8 weeks of age with cardiotoxin in the right gastrocnemius muscle 1 day before injection of 10^6^ U57810 cells in the muscle. Treatment was started once the tumors reached 0.25 cm^3^. Mice were treated with ENT 5 mg/kg/day by intraperitoneal (IP) injection, VCR at a dose of 1 mg/kg weekly by IP injection, or in combination at the same dose and treatment ended when the tumors reached 1.5 cm^3^. During treatment, mice with significant body weight loss approaching (10–15%) were euthanized early per protocol.

### Patient-derived xenograft models at Champions Oncology

The Champions Personalized TumorgraftTM chemosensitivity test was conducted using a TumorGraft model established from a resection of sarcoma, which was removed from the abdomen. “Chemosensitivity” test is used for the patient drug sensitivity testing where we are guiding patients’ clinical therapies by their PDX model responses. In this instance, it is ENT +/− VCR testing. The explant was received and immediately implanted into immunodeficient mice for the purpose of propagating the tumor for the test. All test agents were formulated according to the manufacturer’s specifications. Beginning day 0, tumor dimensions were measured twice weekly by a digital caliper and data, including individual and mean estimated tumor volumes (mean TV ± SEM), are recorded for each group. Tumor volume was calculated using the formula: TV = width^2^ × length × *π*/2. At study completion, percent tumor growth inhibition (%TGI) values were calculated and reported for each treatment group (*T*) versus control (*C*) using initial (*i*) and final (*f*) tumor measurements by the formula: %TGI = [1 − (*Tf* − *Ti*)/(*Cf* − *Ci*)] × 100. Individual mice reporting a tumor volume > 120% of the day 0 measurement are considered to have progressive disease (PD). Individual mice with neither sufficient shrinkage nor sufficient tumor volume increases are considered to have stable disease (SD). Individual mice reporting a tumor volume ≤ 70% of the day 0 measurement for two consecutive measurements over a 7-day period are considered partial responders (PR). If the PR persisted until study completion, percent tumor regression (%TR) is determined using the formula: %TR = (1 − *Tf*/*Ti*) × 100; a mean value is calculated for the entire treatment group. Individual mice lacking palpable tumors for two consecutive measurements over a 7-day period are classified as complete responders (CR). All data collected in this study was managed electronically and stored on a redundant server system. All animal studies were conducted with the approval of Champions Oncology’s International Animal Care and Use Committee (IACUC).

### Patient-derived xenograft models at The Jackson Laboratory

The Jackson Laboratory established each PDX model using NSG (NOD.Cg-*Prkdcscid IL2rgtm1Wjl*/SzJ) mice. Tumor explants obtained from the patients were immediately implanted into the rear flanks of recipient female NSG (JAX # 5557) mice using a trochar. For tumors reaching about 2000 mm^3^, they were collected and passaged for serial transplantation in NSG mice to create low-passage fragments or cohort for future studies. The criteria for enrollment was the tumor volume range of 150–250 mm^3^. Mice were treated with vehicle or ENT/VCR as a single agent or combination at dose and route of administration provided in Additional file 6: Table S1 until tumors reached 2000 mm^3^ or reached study day 28. The antitumor activity of ENT and VCR was tested. All compounds were formulated according to the manufacturer’s specifications. Beginning day 0, tumor dimensions were measured twice weekly by a digital caliper and data including individual and mean estimated tumor volumes (mean TV ± SEM) recorded for each group; tumor volume was calculated using the formula: TV = (width)2 × length/2. All animal studies were conducted with the approval of The Jackson Laboratory IACUC.

### RNAseq

RNA sequencing was performed on four eRMS cultures (human cell lines RD and Rh18, mouse cell cultures U37125 and U57810). To identify transcriptional changes in eRMS following ENT treatment, each sample was treated with ENT for a fixed period of time alongside a paired untreated sample. All samples were treated for with ENT (2 μM) for 72 h. This dose was chosen based on our previous publication [16] where murine rhabdomyosarcoma primary tumor cell cultures were incubated with varying concentration of ENT for 72 h. The cytotoxic effect was then assessed by MTS assay. All cells were cultured on 10-cm dishes, and treatment began when plates were 60% confluent. Passages lower than 7 were used for all mouse cultures. Bioinformatic analysis of RNAseq performed as described in our earlier study [14].

### CRISPR screening in eRMS

#### Cell culture, sgRNA designs, and virus production

Murine eRMS tumor cell line (U57810) expressing hCas9 was derived from retroviral transduction of MSCV-hCas9-PGK-Puro vector into a parental U57810 cell line, followed by puromycin selection (1 μg/mL). All sgRNAs were designed using http://crispr.mit.edu/ with high-quality scores (> 70) to minimize off-target effects and were subsequently cloned into U6-sgRNA-EFS-GFP construct. HDAC sgRNAs were designed to specifically target the deacetylase catalytic domains as previously described [17]. *Rosa26 and Rpa3* sgRNAs were used as negative and positive controls, respectively. Lentiviral sgRNA constructs were transfected together with viral packaging vectors (pPAX2: VSVG) using the standard protocol for PEI reagent (23966, Polysciences) into HEK293T cells. Viral supernatants were collected between 24 to 48 h post-transfection and passed through a 0.45-μm filter.

### sgRNA/GFP competition assays

To evaluate sgRNA effects on eRMS cell proliferation, U57810 expressing Cas9 cells were transduced with sgRNA virus, followed by flow cytometry analysis of GFP/sgRNA+ populations using a Guava Easycyte HT instrument (Millipore) over a course of 16 days after viral infection. GFP percentages at indicated time points on histograms were normalized to day 2 GFP percentages post-infection.

### Statistical analysis

The statistical test used has been described in our previous study [14]. Briefly, in the case of the orthotopic mouse model of murine eRMS, failure is defined as an event for tumor size greater than or equal to 1.2 cm^3^. Treatment groups were contrasted on the mean with analysis of variance in log units. Time to event distributions were summarized with Kaplan-Meier curves, and the significance of variation with the treatment group was assessed with log-rank tests. Corrections for multiple comparisons were made with the Dunnett method for ANOVA and with the Bonferroni method for log-rank testing. Statistical testing on means and time to event was two-sided with a nominal significance level of 5% and was carried out with R. The significance of variation intumor volume with treatment was assessed in PDX models using a repeated-measureslinear model with an autoregressive order 1 autocorrelation matrix and a Tukey correction for multiple comparisons in terms of treatment, day, and the treatment × day interaction. All statistical testing was two sidedwith a 5% experiment-wise significance level and all analyses were carried out in log10 units. SAS version 9.4 for Windows (SAS Institute) was used throughout. Statistical significance was set at *P < 0.05, **P < 0.01, and ***P < 0.001. Error bars indicate mean ± SD or SEM.

## Results

### ENT in combination with vincristine slows eRMS tumor growth and induces myodifferentiation in vivo

**Fig. 1:**
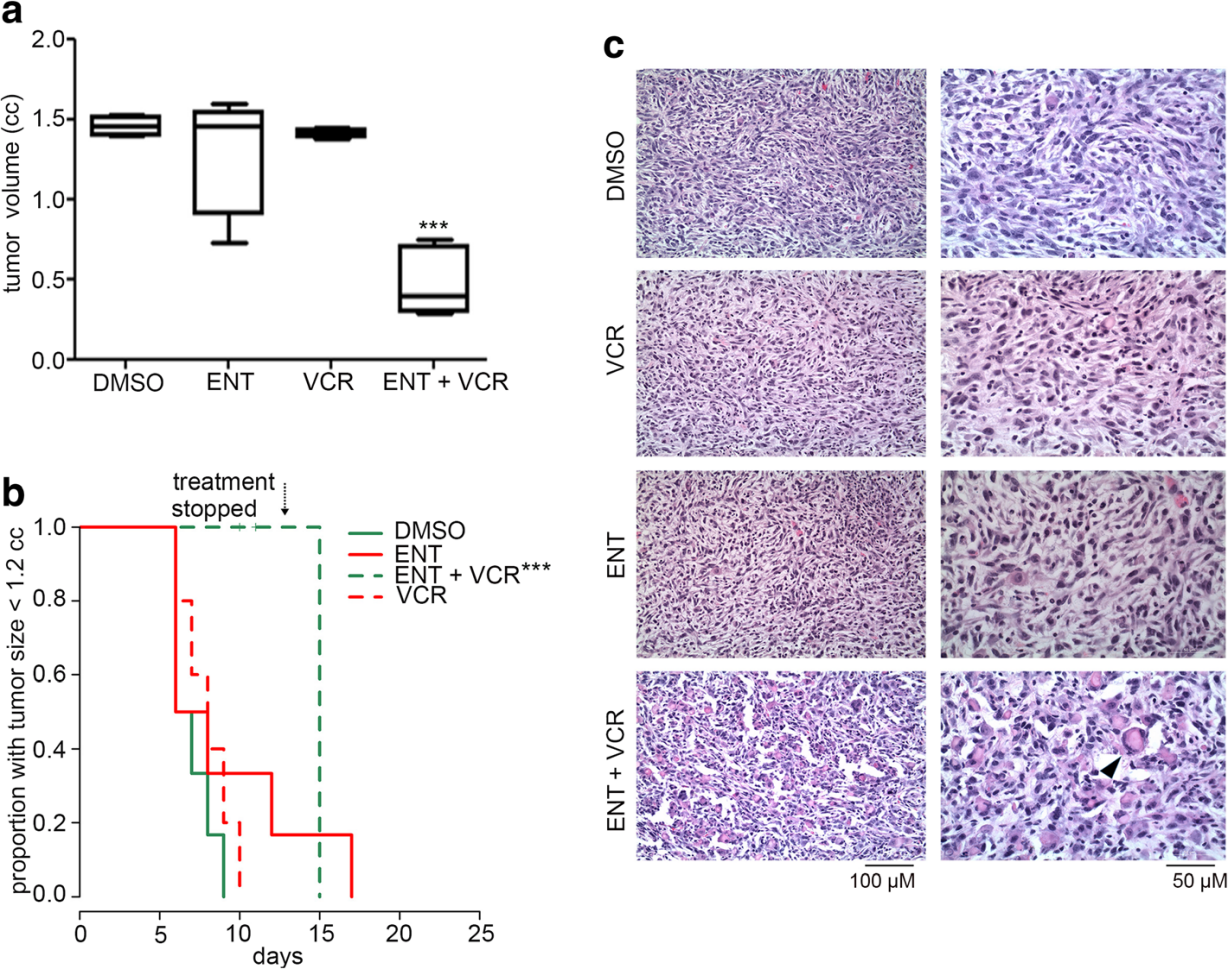
ENT treatment of eRMS in vivo. **a** Box-and-whisker plots showing the tumoristatic efficacy of entinostat in combination with vincristine in eRMS mice (DMSO vs Ent + VCR. Data are presented as means ± SEM (*N* = 5 mice per cohort). ***P < 0.001 by log-rank test. **b** Kaplan-Meier plots of eRMS mice treated with ENT at a daily dose of 5 mg/kg administered intraperitoneally, VCR at 1 mg/kg administered intraperitoneally once a week or a combination of both. Treatment was stopped early for all mice after day 11 because of body weight loss in some of the animals. Data are presented as means ± SEM (*N* = 5 mice per cohort). ***P < 0.001 by log-rank test. **c** Samples were collected from mice upon necropsy at study completion. Tumors were stained by hematoxylin and eosin and scored blindly. Rhabdomyoblasts are visible in ENT + VCR treated cells (pointed with white arrowhead). Scale bar, 100 μM

We tested the efficacy of ENT and VCR as single agents and in combination in orthotopic allograft mouse models of eRMS. eRMS does not harbor Pax3:Foxo1 fusion, and thus, response was not expected to mimic fusion-positive RMS [14]. The eRMS model was generated by injecting murine eRMS primary cell cultures into the cardiotoxin-preinjured gastrocnemius muscle of SHO mice. ENT as a single agent showed minimal antitumor activity in this embryonal model of RMS; however, in these fusion-negative (Pax3:Foxo1 non-expressing) mice, treatment with the combination of ENT plus VCR reduced tumor volume significantly (Fig. 1a, b).

Since combined treatment with ENT and VCR caused a reduction in volumes of eRMS tumors (despite not carrying *Pax3:Foxo1* fusion gene), we investigated whether treatment with ENT and VCR contributed to rhabdomyoblastic differentiation. Residual end-treatment tumors were evaluated histologically, and rhabdomyoblastic differentiation was scored for all the four treatment groups. The percentage of rhabdomyoblasts in the tumors from mice treated with the combination of ENT and VCR was prominent (83% rhabdomyoblasts on average) compared to mice treated with ENT alone (21%) or VCR alone (20%). Representative histology of each treatment group is provided in Fig. 1c. Mice bearing eRMS treated with the combination of VCR and ENT had very few mitotic figures (3.5% mitotic figures on average) in comparison to mice treated with VCR only (12%)—suggesting the rhabdomyoblasts were quiescent or non-proliferative rhabdomyoblasts (Additional file 1: Figure S1a-c). These studies supported that eRMS tumor cells that escaped cytoreduction were subject to rhabdomyoblastic differentiation—but only when ENT is combined with a specific chemotherapeutic agent (VCR). This result is consistent with clinical observations of rhabdomyoblastic differentiation induced by treatment stress [18].

### ENT has single-agent activity in eRMS patient-derived xenografts

**Fig. 2:**
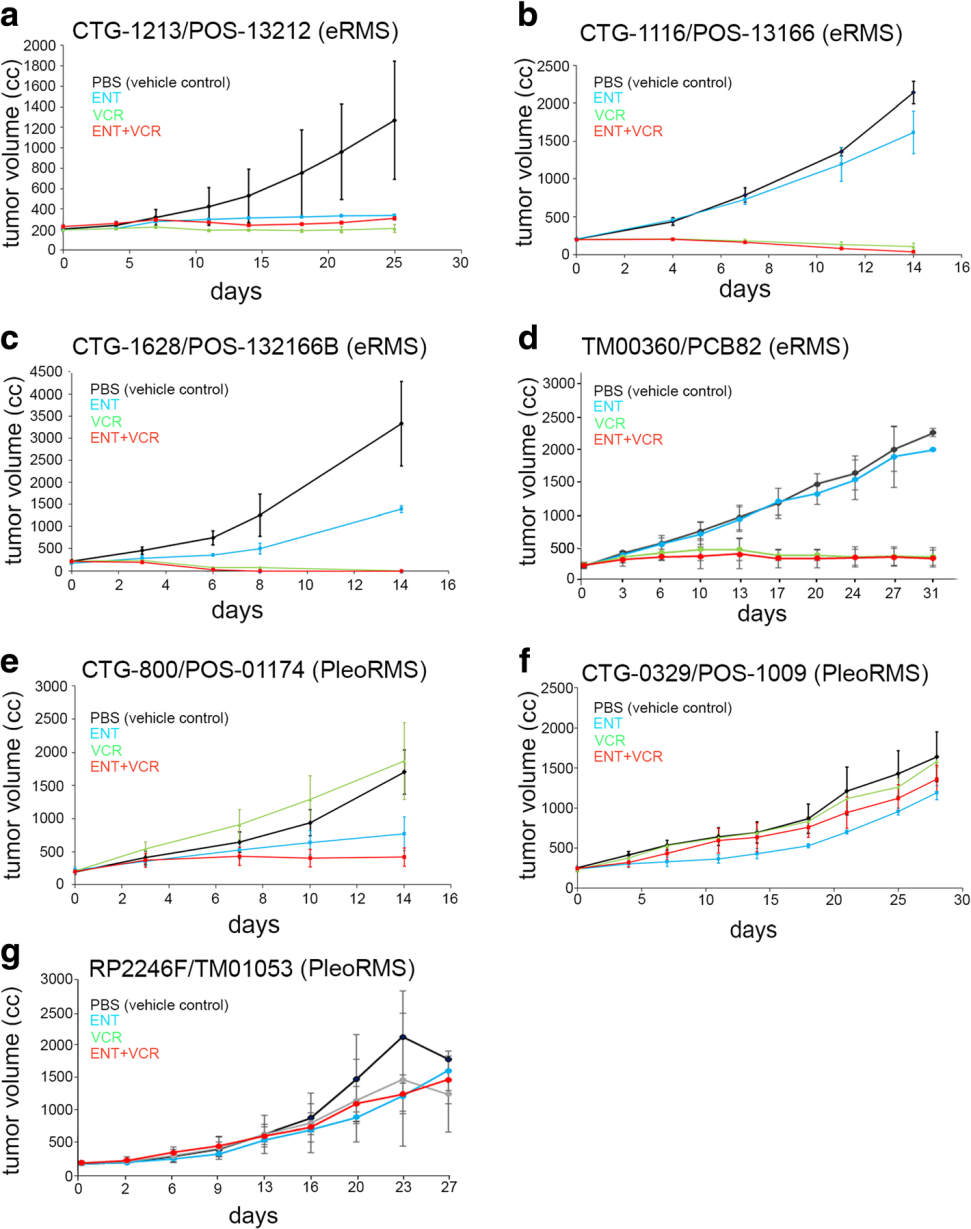
In vivo evaluation of ENT and VCR in eRMS and pleoRMS patient-derived xenograft. **a–g** Graphical analysis of four different PDX eRMS and three different PDX pleoRMS mice models (Champions Oncology and The Jackson Laboratory) established from clinical biopsies, recurrent eRMS, or autopsies male/female with eRMS. Detailed patient history of these eRMS and pleoRMS models is given in Additional file 6: Table S2 & S8 respectively. Mice were treated with vehicle, ENT (4 mg/kg or 5 mg/kg), and vincristine (0.75 mg/kg) as single agents and in combination in each treatment groups and average tumor volume were plotted until the end-point of the experiment. Detailed treatment schedules are given in Additional file 6: Table S1. Statistical summary for the response to treatment is given in Additional file 6: Table S4–S6 for eRMS PDX models and in Additional file 6: Table S9–S11 for pleoRMS. Effectiveness of VCR precluded drawing any conclusions of ENT-VCR combination therapy in eRMS models

We investigated the antitumor efficacy of ENT and VCR as single agents and in combination for four biologically independent patient-derived xenograft mouse models of eRMS. The dosing details are given in Additional file 6: Table S1a-b, and the PDX model characteristics are given in Additional file 6: Table S2. Two of the four models were from recurrent and/or metastatic tumors taken from a biopsy or rapid autopsy. All of these contemporary models were established after 2010. In half of the cases, ENT showed single-agent activity in tumor growth inhibition relative to control (Fig. 2a–d). These eRMS models were hypersensitive to the VCR dose used in aRMS PDX models; thus, the synergy between ENT and VCR could not be assessed in this study. Statistical summaries of four different PDX eRMS are given in Additional File 6: Table S2-S6. Residual end-treatment tumors were examined histologically, and rhabdomyoblastic differentiation was scored for the CTG-1213 PDX mouse model. No difference existed between different treatment groups in terms of rhabdomyoblast differentiation except for combination (ENT + VCR) showing moderate differentiation (20%) (Additional file 6: Table S7).

### ENT has single-agent activity in PleoRMS patient-derived tumorgraft xenografts

We next investigated the antitumor efficacy of ENT and VCR as single agents and in combination in three biologically independent patient-derived tumorgraft xenograft mouse models of pleoRMS. PDX model characteristics are given in Additional file 6: Table S8. In all the cases, ENT had single-agent activity relative to control (Fig. 2e–g). Statistical summaries of three different PDX pleoRMS models are given in Additional file 6: Table S9-S11. Residual end-treatment tumors were examined histologically, and rhabdomyoblastic differentiation was scored for the CTG-800 PDX mouse model, which showed the best response to treatment. There was no difference seen between different treatment groups in terms of rhabdomyoblast differentiation (Additional file 6: Table S12). Representative histology of each treatment group is provided in Additional file 2: Figure S2.

### HDAC3 inhibition is responsible for tumor cell growth inhibition in eRMS

**Fig. 3:**
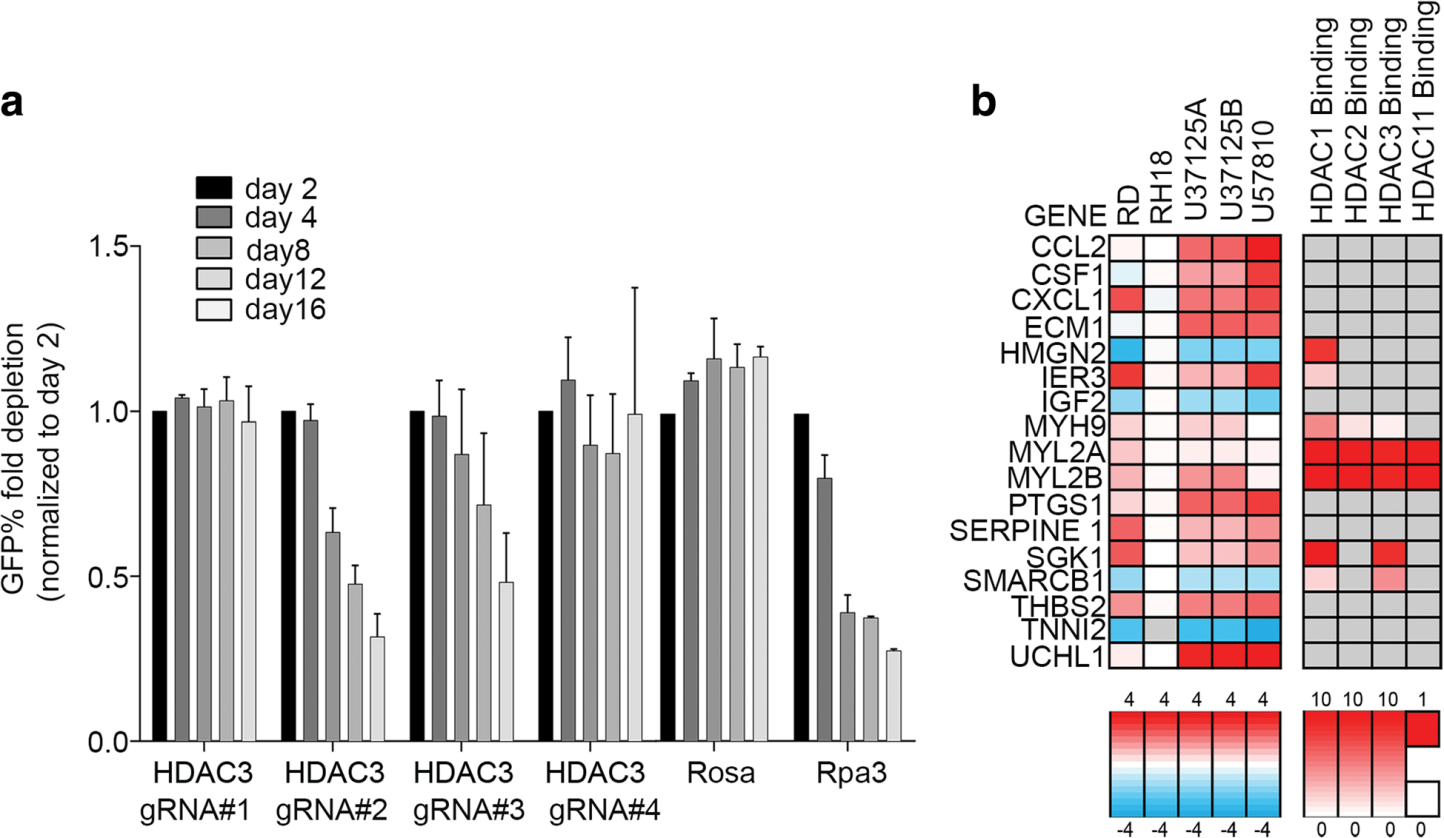
CRISPR/Cas9 mediated HDAC3 inhibition and evaluation of tumor cell growth inhibition in eRMS and HDAC1/2/3/11 binding data for key ENT-treated eRMS samples. **a** CRISPR/Cas9 screen for the viability of selected HDAC3 excision by CRISPR in murine eRMS. **b** RNA from four human and murine eRMS cultures (U37125 in two replicates, U57810, RD, RH18) were sequenced following ENT treatment at 2 μM for 72 h and compared against DMSO-treated cultures. Rh18 which was hypersensitive to ENT and was only treated for 24 h. Key hits were identified as those overexpressed (log2 ratio of ENT expression divided by control expression > 1) in all samples

Our recent study showed HDAC3 inhibition by ENT as the primary mechanism in aRMS [14]. To determine whether inhibition of a specific HDAC3 target of ENT was responsible for cytoreduction in eRMS, we performed CRISPR-Cas9-mediated targeting of the deacetylase domains of HDAC3 in the U57810 murine eRMS primary tumor cell culture which were then tracked for cell viability over 16 days. The HDAC3 CRISPR constructs reduced the viability of eRMS cells (Fig. 3a) whereas other HDAC CRISPR did not reduce the cell viability (Additional file 3: Figure S3a-d). These results suggest that in eRMS, HDAC3 is a cell-autonomous survival factor. We have previously performed a cell viability assay in murine eRMS culture, U57810 (in which we carried out CRISPR-mediated depletion of HDAC3), using entinostat. In U57810, a dose-dependent change in cell viability at 72 h is present (IC25 1.2 μM; IC50 3.9 μM) (data not shown). By comparison, CRISPR HDAC3 depletion in the same cell line showed a minimal effect at day 4 and a maximum effect at day 16. These data are consistent and argue for a prolonged time-dependent effect on cell viability in the cell-autonomous experimental context.

To determine whether myogenic differentiation was a cell-autonomous effect of ENT-VCR combination therapy in eRMS, U57810 cells were treated with VCR and ENT individually and in combination for 6 days. RT-PCR measuring the mRNA expression of four different muscle differentiation markers showed no apparent differentiation effect in vitro (Additional file 3: Figure S3e). In addition, we performed siRNA-mediated knockdown of HDAC3 in eRMS cell line RD and analyzed for the protein expression of terminal differentiation marker, myosin heavy chain (MHC). We used differentiated human myoblasts as the positive control. Analysis of MHC expression using anti-My3 antibody showed MHC expression in the lane for differentiated HSMM. However, we did not detect MHC expression in RD cells depleted of HDAC3. In addition to eRMS cell line, we also analyzed aRMS cell line Rh30 depleted of HDAC3 for MHC expression. The result is similar to the eRMS cell line with no induced expression of MHC upon depletion of HDAC3 (Additional file 4: Figure S4). This is in contradistinction to the observed significant in vivo change in rhabdomyoblast counts for this murine eRMS model. This difference between in vitro and in vivo results raises the possibility that species-specific tumor microenvironment factors are critical to the myodifferentiation effect observed in vivo factors that may have been present in allografts but not xenografts.

### Transcriptional reprogramming by ENT differs for aRMS versus eRMS

We next turned to short-term RNAseq in order to explore whether ENT induced tumor cell production of factors related to remodeling the tumor microenvironment. We have previously shown that tumor cells can interact with muscle stem cells (i.e., satellite cells) in an IL-4R-dependent manner to accelerate tumor progression [19]. An integrated dataset was constructed for eRMS consisting of differential expression data and HDAC1, 2, 3, or 11 binding data (Fig. 3b). This merged dataset identified several genes of interest. To understand the broad changes in the transcriptional program for eRMS due to ENT treatment, Gene Ontology analysis [20] was carried out using PANTHER version 14.0 to identify key upregulated or downregulated processes. This ontology analysis of eRMS identified several key upregulated muscular programs: muscle contraction, muscle system process, muscle organ development, muscle structure development, and cell differentiation (Additional file 7: Table S14), which surprisingly does not translate into in vitro myodifferentiation as seen in vivo with the possibility that tumor microenvironment factors are critical to the myodifferentiation effect observed in vivo in response to ENT treatment.

However, more prominent than any other feature of ENT-regulated genes was the cohort of cytokine (CCL2, CSF1, CXCL1), growth factor (IGF2), and extracellular matrix genes (ECM1, SERPINE1) (Fig. 3b). These factors are associated with both myoblast/myofiber communication and differentiation [21] and macrophage interactions [22]. However, when re-examining ENT-VCR-treated mice, macrophage infiltration was increased only in areas of necrosis, not elsewhere. No difference in macrophage infiltration was observed between treatment groups (Additional file 5: Figure S5). Taken together, these results suggest that following cell-autonomous tumor cell death caused by HDAC3 inhibition, residual tumor cells under chemotherapeutic stress are induced to express cytokines and growth factors. These secreted factors may in turn lead to non-macrophage tumor microenvironment interactions facilitating rhabdomyoblastic differentiation of adjacent tumor cells.

## Discussion

Our previous study uncovered that cell of origin epigenetically influenced transcription of the *PAX3:FOXO1* oncogene in fusion-positive RMS [16]. This observation led to the investigation of pharmacological modifiers of transcription of the fusion protein via HDAC inhibition. ENT, a potent and selective Class I HDAC inhibitor, reduced the abundance of the tumor-driving *PAX3:FOXO1* mRNA and protein expression [14]. Further investigation revealed HDAC3-SMARCA4-miR-27a-PAX3:FOXO1 circuit as a critical driver of chemo-resistant fusion-positive RMS [14]. The purpose of the current study was to investigate the preclinical efficacy of ENT in the other and more common subtype, eRMS [23]. ENT in combination with the chemotherapy agent VCR showed strong antitumor activity in eRMS orthotopic mouse models, antitumor activity in eRMS PDX models as a single agent in half of the cases, and a range of single-agent activity in pleoRMS PDX models. Although our in vitro concentration of ENT was higher at 400 nM and 2000 nM, our preclinical dose in eRMS and pleoRMS PDX models were clinically comparable (or slightly under-dosed) and similar to our recent study of ENT in aRMS [14]. In addition, the concentration of 400 nM used in vitro was less than the reported highest achievable maximum drug serum concentration (C_max_) for ENT of 1000 nM [24], and the 2000 nM concentration was comparable to the very maximum dose of ENT used in our previous study [14] to see a dose-dependent effect of ENT in fusion-positive RMS.

Our CRISPR studies of the HDAC targets of ENT suggest that HDAC3 is a key factor to eRMS cell-autonomous tumor cell survival. Following cytoreduction in vivo in orthotopic allografts, residual tumor cells treated with chemotherapy undergo a dramatic, ENT-induced (70–100%) conversion to non-proliferative rhabdomyoblasts, raising the possibility that myogenic differentiation could be a true therapeutic goal [25]. Rhabdomyoblasts remaining at the end of multimodal therapy rarely if ever lead to disease recurrence and may truly represent a differentiated state [26]. Our studies further narrow the mechanism of this effect to the potential interplay of tumor cells secreting cytokines and non-malignant cells and the tumor microenvironment to cause this tumor cell differentiation. In eRMS PDX models, cytoreduction was a consistent feature, but myogenic differentiation was noticeably absent. This result is in contrast to the recently published work, which showed that HDAC3 knockout arrests tumor growth and induces myogenic differentiation both in vitro and in vivo [27].

## Conclusion

In summary, our data suggest that targeting Class I HDACs may provide a therapeutic benefit for patients with eRMS. The single agent preclinical in vivo efficacy of ENT begets exploration of a combination with chemotherapy in a clinical trial for eRMS—although our studies of PDX models had too strong of VCR single-agent activity to detect synergy. The synergy in vivo of ENT in combination with chemotherapy for fusion-negative eRMS orthotopic allograft models and unexpected myodifferentiation effect brings new possibilities that epigenetic modifiers can not only cytoreduce tumors but also reprogram cancer cells towards a non-tumorigenic cell fate as a desired therapeutic outcome.

## Additional files

Additional file 1: **Figure S1.** Phosphohistone H3 (pHH3) expression and rhabdomyoblast count in VCR and ENT + VCR-treated eRMS. (a) pHH3 expression of eRMS mouse tumor (animal id: 67833) after treatment with single agent VCR. Scale Bar: 50 μM. (b) pHH3 expression of eRMS mouse tumor (animal id: 67822) after treatment with both ENT and VCR. Scale bar: 50 μM (c) Histological scoring of various mouse eRMS after four different types of treatment; DMSO, VCR, ENT, or a combination of ENT and VCR (ENT + VCR). Counted all fields 500 to 3000 cells under low and higher magnification on each slide. This count excluded endothelial cells and inflammatory cells. * denotes not determined while ** denotes myoglobin was immunohistochemically positive. (TIF 2381 kb)

Additional file 2: **Figure S2.** Representative histology of CTG-800 PDX mouse pleoRMS rhabdomyosarcoma tissue. Tumors were stained by hematoxylin and eosin and scored blindly. Scale Bar, 100 μM. (TIF 2568 kb)

Additional file 3: **Figure S3.** CRISPR/Cas9 mediated HDACs inhibition and evaluation of tumor cell growth inhibition in eRMS. (a-e) CRISPR/Cas9 screen for viability of selected HDACs (HDAC1-2 & HDAC10-11) excision by CRISPR in murine eRMS. (f) Q-PCR of murine eRMS U57810 for the expression of myogenic markers of differentiation in vitro. Data normalized to GAPDH expression. Gene expression was quantified using 2^−^dCt^ method. Myogenin (MYOG), Myoblast determination protein 1 (MyoD) and Myoglobin (Mb). (TIF 2070 kb)

Additional file 4: **Figure S4.** siRNA-mediated knockdown of HDAC3 in eRMS and aRMS. Analysis of MHC expression in RD, Rh30 and HSMM cell lines transfected with siRNA at 100 nM for 72 h, targeting HDAC3. (TIF 510 kb)

Additional file 5: **Figure S5.** Representative immunohistochemistry for CD68 of mouse eRMS tissue and primary cells. Necrotic tissue (a) showed few macrophages present, while viable tumor (b) showed a collection of many macrophages. Macrophage presence was observed as being the same for all treatment groups. (TIF 2929 kb)

Additional file 6: **Table S1.** Treatment schedule for PDX models. **Table S2.** Patient history of PDX eRMS models. **Table S3.** Statistical summary for CTG-1213/POS-13212. **Table S4.** Statistical summary for CTG1116/POS-13166. **Table S5.** Statistical summary for CTG-1628/POS-132166B. **Table S6.** Statistical summary for J0103366/CF-13A. T**able S7.** Histological markers of differentiation in PDX eRMS (CTG-1213) mice. **Table S8.** Patient history of PDX pleoRMS models.** Table S9.** Statistical summary for CTG-1213/POS-13212. **Table S10.** Statistical summary for CTG1116/POS-13166. **Table S11.** Statistical summary for CTG-1628/POS-132166B. **Table S12.** Histological markers of differentiation in PDX pleoRMS (CTG-800) mice. **Table S13.** Primers for RT-PCR. (DOCX 41 kb)

Additional file 7: **Table S14.** Gene ontology analysis. (XLSX 62 kb)

## Abbreviations

aRMS: Alveolar rhabdomyosarcoma
C_max_: Maximum drug serum concentration
ENT: Entinostat
eRMS: Embryonal rhabdomyosarcoma
HDAC: Histone deacetylase
IP: Intraperitonial
PDX: Patient-derived xenografts
PR: Partial response
RMS: Rhabdomyosarcoma
SD: Stable disease
SHO: SCID Hairless Outbred
VCR: Vincristine
