# Supplementary figures and images for: Preclinical rationale for entinostat in embryonal rhabdomyosarcoma
Source: Skelet Muscle. 2019 May 21;9:12. doi: 10.1186/s13395-019-0198-x (PMC6528217; doi:10.1186/s13395-019-0198-x)

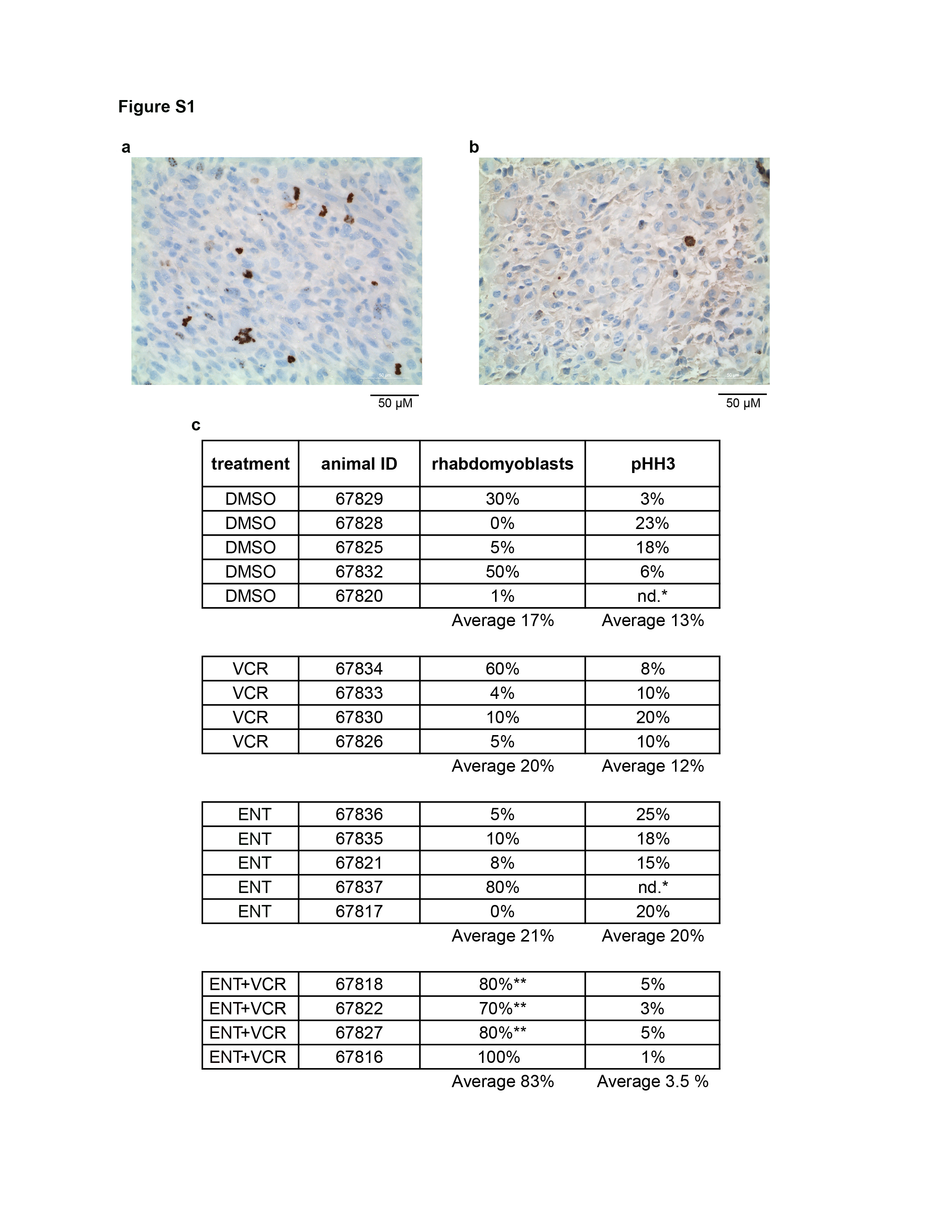

Supplement: Supplementary file 1 — Figure S1. Phosphohistone H3 (pHH3) expression and rhabdomyoblast count in VCR and ENT + VCR-treated eRMS. (a) pHH3 expression of eRMS mouse tumor (animal id: 67833) after treatment with single agent VCR. Scale Bar: 50 μM. (b) pHH3 expression of eRMS mouse tumor (animal id: 67822) after treatment with both ENT and VCR. Scale bar: 50 μM (c) Histological scoring of various mouse eRMS after four different types of treatment; DMSO, VCR, ENT, or a combination of ENT and VCR (ENT + VCR). Counted all fields 500 to 3000 cells under low and higher magnification on each slide. This count excluded endothelial cells and inflammatory cells. * denotes not determined while ** denotes myoglobin was immunohistochemically positive. (TIF 2381 kb) [file 13395_2019_198_MOESM1_ESM.tif]

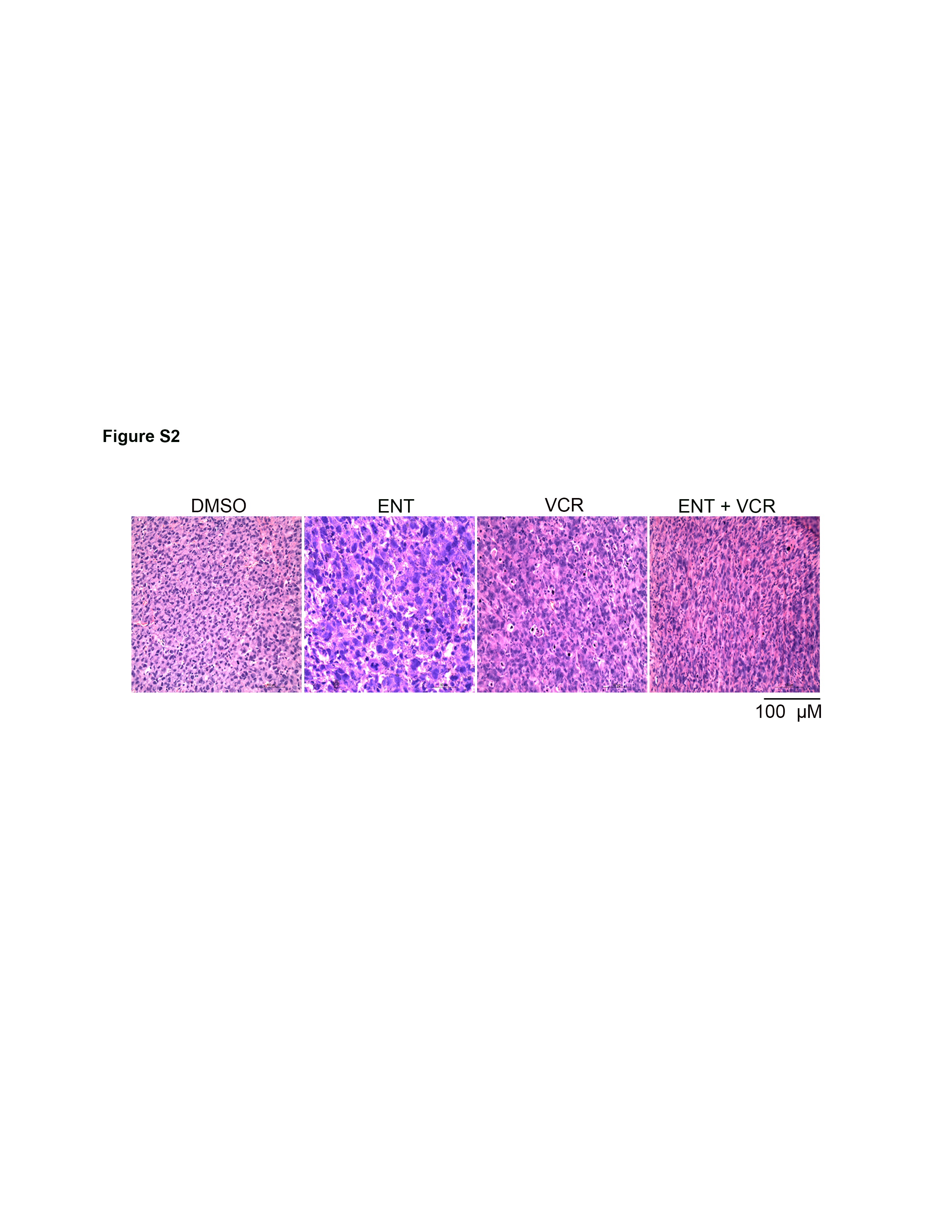

Supplement: Supplementary file 2 — Figure S2. Representative histology of CTG-800 PDX mouse pleoRMS rhabdomyosarcoma tissue. Tumors were stained by hematoxylin and eosin and scored blindly. Scale Bar, 100 μM. (TIF 2568 kb) [file 13395_2019_198_MOESM2_ESM.tif]

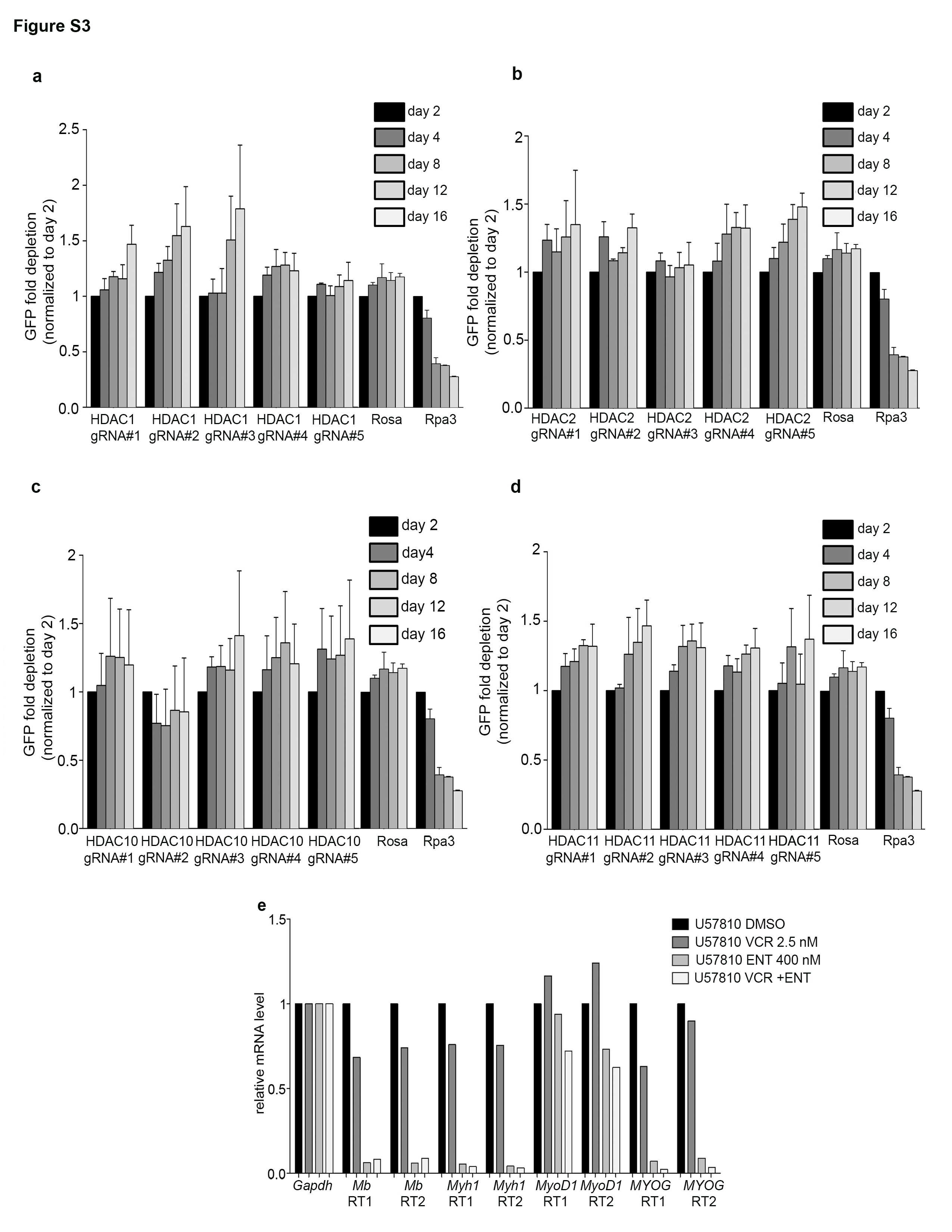

Supplement: Supplementary file 3 — Figure S3. CRISPR/Cas9 mediated HDACs inhibition and evaluation of tumor cell growth inhibition in eRMS. (a-e) CRISPR/Cas9 screen for viability of selected HDACs (HDAC1-2 & HDAC10-11) excision by CRISPR in murine eRMS. (f) Q-PCR of murine eRMS U57810 for the expression of myogenic markers of differentiation in vitro. Data normalized to GAPDH expression. Gene expression was quantified using 2−^dCt method. Myogenin (MYOG), Myoblast determination protein 1 (MyoD) and Myoglobin (Mb). (TIF 2070 kb) [file 13395_2019_198_MOESM3_ESM.tif]

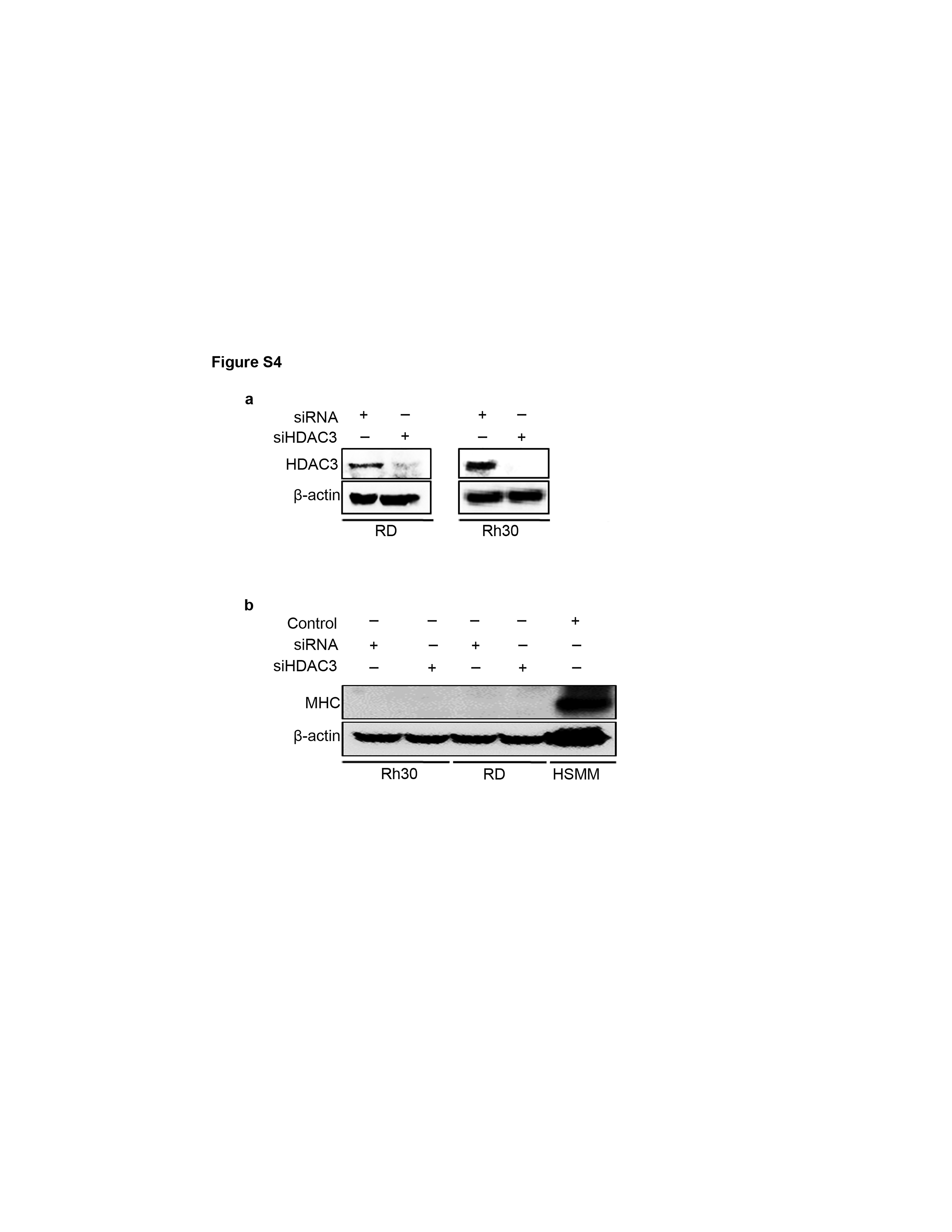

Supplement: Supplementary file 4 — Figure S4. siRNA-mediated knockdown of HDAC3 in eRMS and aRMS. Analysis of MHC expression in RD, Rh30 and HSMM cell lines transfected with siRNA at 100 nM for 72 h, targeting HDAC3. (TIF 510 kb) [file 13395_2019_198_MOESM4_ESM.tif]

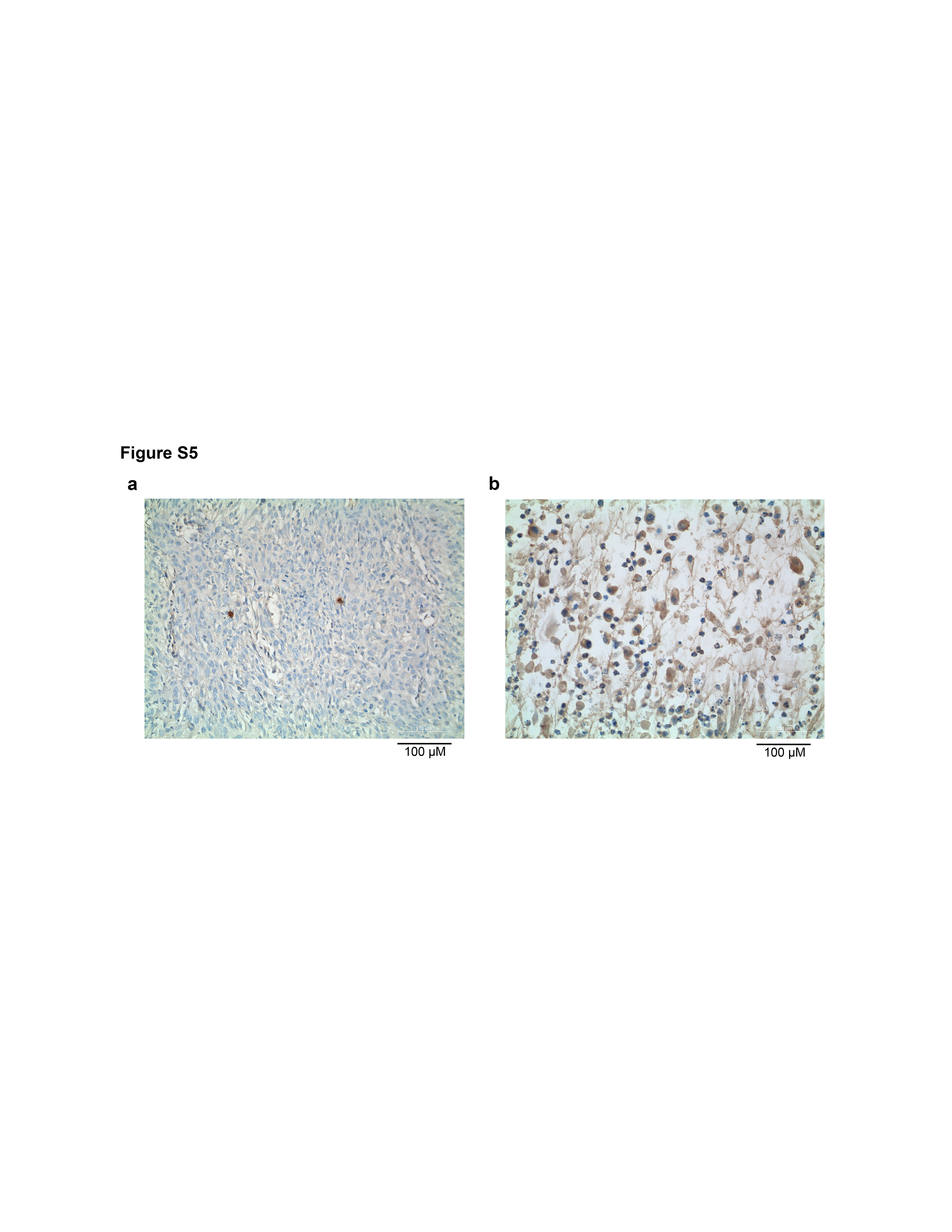

Supplement: Supplementary file 5 — Figure S5. Representative immunohistochemistry for CD68 of mouse eRMS tissue and primary cells. Necrotic tissue (a) showed few macrophages present, while viable tumor (b) showed a collection of many macrophages. Macrophage presence was observed as being the same for all treatment groups. (TIF 2929 kb) [file 13395_2019_198_MOESM5_ESM.tif]
