# Supplementary material for: Preclinical rationale for entinostat in embryonal rhabdomyosarcoma
Source: Skelet Muscle. 2019 May 21;9:12. doi: 10.1186/s13395-019-0198-x (PMC6528217; doi:10.1186/s13395-019-0198-x)
Supplement: Supplementary file 6 — Table S1. Treatment schedule for PDX models. Table S2. Patient history of PDX eRMS models. Table S3. Statistical summary for CTG-1213/POS-13212. Table S4. Statistical summary for CTG1116/POS-13166. Table S5. Statistical summary for CTG-1628/POS-132166B. Table S6. Statistical summary for J0103366/CF-13A. Table S7. Histological markers of differentiation in PDX eRMS (CTG-1213) mice. Table S8. Patient history of PDX pleoRMS models. Table S9. Statistical summary for CTG-1213/POS-13212. Table S10. Statistical summary for CTG1116/POS-13166. Table S11. Statistical summary for CTG-1628/POS-132166B. Table S12. Histological markers of differentiation in PDX pleoRMS (CTG-800) mice. Table S13. Primers for RT-PCR. (DOCX 41 kb) [file 13395_2019_198_MOESM6_ESM.docx]

**Table S1. Treatment Schedule for the PDX mice.** Treatment schedules for the

Champions Oncology (A) and Jackson Laboratory (B) PDX model mice, including the

group of mice, number of mice, treatment agent used, doses and drug administration

schedule given. p.o., oral; i.p., intraperitoneal; qd, daily; bid, twice a day; q7d, every 7

days.

**a. Jackson Laboratories treatment schedule**

| Group | n | Agent | Dose (mg/kg/dose) | ROA/Schedule |
| --- | --- | --- | --- | --- |
|  |  |  |  |  |
| 1 | 3 | control |  |  |
| 2 | 3 | ENT | 4 | p.o./qdx21 |
| 3 | 3 | VCR | 0.75 | i.p./q7dx3 |
| 4 | 3 | ENT  VCR | 4  0.75 | p.o./qdx21 i.p./q7dx3 |

**b. Champions treatment schedule**

| Group | n | Agent | Dose (mg/kg/dose) | ROA/Schedule |
| --- | --- | --- | --- | --- |
|  |  |  |  |  |
| 1 | 2 | control |  |  |
| 2 | 2 | ENT | 5 | p.o./qdx21 |
| 3 | 2 | VCR | 0.75 | i.p./q7dx3 |
| 4 | 2 | ENT  VCR | 5  0.75 | p.o./qdx21  i.p./q7dx3 |

**Table S2. Patient history of PDX eRMS models**

| **RMS Model** | **Alias** | **Histology** | **Harvest site** | **Mutations** | **Disease stage** | **Time of Biopsy** | **Age** | **Gender** |
| --- | --- | --- | --- | --- | --- | --- | --- | --- |
| CTG-1213 | POS-13212 | embryonal | vagina | NRAS [Q61K], BCL9 [H1105], CEBPA [X197X] | n/a | first diagnosis | 2 | F |
| CTG-1116 | POS-13166 | embryonal | neck | EML4 [V5881], FANCA [G106A], PIK3CA [E545K], SEPT6 [ A128S] | n/a | first diagnosis | 6 | M |
| CTG-1628 | POS-13166B | embryonal | neck | ASXL1, BRCA1 [1690S]  IGF2:ARID1A | n/a | recurrent | 7 | M |
| PCB82^#^ | SA0082F/TM00360 | embryonal | parameninges (autopsy) | AURKA [F31I], EHMT2 [T55N], TP53 [P72R] RET [G691S] | IV | Autopsy | 14 | F |

^#^ nb. The ALK R1181C mutation reported from the biopsy for PCB82 [1] was not present in the PDX model.

**Table S3: Statistical summary for CTG-1213/POS-13212**

| *day* | *group* | *comparator* | *difference* | *raw CI* | *raw p value* | *tukey CI* | *tukey p value* |
| --- | --- | --- | --- | --- | --- | --- | --- |
| 25 | Control | ENT | 0.46±0.2 | (0.06, 0.86) | 0.03 | (-0.36, 1.28) | 0.85 |
| 25 | Control | ENT/VCR | 0.5±0.2 | (0.1, 0.91) | 0.02 | (-0.32, 1.33) | 0.73 |
| 25 | Control | VCR | 0.69±0.2 | (0.28, 1.09) | 0.002 | (-0.14, 1.51) | 0.21 |
| 25 | ENT | ENT/VCR | 0.04±0.2 | (-0.36, 0.44) | 0.83 | (-0.78, 0.86) | 1 |
| 25 | ENT | VCR | 0.22±0.2 | (-0.18, 0.62) | 0.26 | (-0.6, 1.04) | 1 |
| 25 | ENT/VCR | VCR | 0.18±0.2 | (-0.22, 0.58) | 0.36 | (-0.64, 1) | 1 |

**Table S4: Statistical summary for CTG1116/POS-13166**

| *day* | *group* | *comparator* | *difference* | *raw CI* | *raw p value* | *tukey CI* | *tukey p value* |
| --- | --- | --- | --- | --- | --- | --- | --- |
| 14 | Control | ENT | 0.13±0.15 | (-0.19, 0.46) | 0.4 | (-0.51, 0.78) | 1 |
| 14 | Control | ENT/VCR | 1.8±0.15 | (1.47, 2.13) | <0.001 | (1.15, 2.44) | <0.001 |
| 14 | Control | VCR | 1.4±0.15 | (1.07, 1.72) | <0.001 | (0.75, 2.04) | <0.001 |
| 14 | ENT | ENT/VCR | 1.67±0.15 | (1.34, 1.99) | <0.001 | (1.02, 2.31) | <0.001 |
| 14 | ENT | VCR | 1.26±0.15 | (0.93, 1.59) | <0.001 | (0.62, 1.91) | <0.001 |
| 14 | ENT/VCR | VCR | -0.4±0.15 | (-0.73, - 0.07) | 0.02 | (-1.05, 0.24) | 0.54 |

**Table S5: Statistical summary for CTG-1628/POS-132166B**

| *day* | *group* | *comparator* | *difference* | *raw CI* | *raw p value* | *tukey CI* | *tukey p value* |
| --- | --- | --- | --- | --- | --- | --- | --- |
| 14 | Control | ENT | 18.97±5.97 | (6.31, 31.63) | 0.006 | (-5.93, 43.87) | 0.26 |
| 14 | Control | ENT/VCR | 56.03±5.97 | (43.37, 68.69) | <0.001 | (31.13, 80.93) | <0.001 |
| 14 | Control | VCR | 54.22±5.97 | (41.56, 66.88) | <0.001 | (29.32, 79.12) | <0.001 |
| 14 | ENT | ENT/VCR | 37.06±5.97 | (24.4, 49.72) | <0.001 | (12.16, 61.96) | 0.001 |
| 14 | ENT | VCR | 35.26±5.97 | (22.6, 47.92) | <0.001 | (10.36, 60.16) | 0.002 |
| 14 | ENT/VCR | VCR | -1.8±5.97 | (-14.46, 10.86) | 0.77 | (-26.7, 23.1) | 1 |

**Table S6: Statistical summary for TM00360/PCB82**

| *day* | *group* | *comparator* | *difference* | *raw CI* | *raw p value* | *tukey CI* | *tukey p value* |
| --- | --- | --- | --- | --- | --- | --- | --- |
| 31 | Control | ENT | 0.04±0.13 | (-0.22, 0.31) | 0.74 | (-0.5, 0.59) | 1 |
| 31 | Control | ENT/VCR | 0.93±0.12 | (0.69, 1.18) | <0.001 | (0.44, 1.43) | <0.001 |
| 31 | Control | VCR | 0.9±0.12 | (0.66, 1.14) | <0.001 | (0.41, 1.39) | <0.001 |
| 31 | ENT | ENT/VCR | 0.89±0.13 | (0.63, 1.15) | <0.001 | (0.36, 1.42) | <0.001 |
| 31 | ENT | VCR | 0.86±0.13 | (0.59, 1.12) | <0.001 | (0.32, 1.39) | <0.001 |
| 31 | ENT/VCR | VCR | -0.03±0.12 | (-0.27, 0.2) | 0.77 | (-0.51, 0.45) | 1 |

**Table S7: Histological markers of differentiation in PDX eRMS (CTG-1213) mice**

| Serial number of eRMS tumor (PDX) type | Percentage rhabdomyoblasts | Treatment |
| --- | --- | --- |
| 184434 | 0 | DMSO |
| 184435 | 0 | DMSO |
| 184442 | 0 | ENT |
| 184450 | 0 | ENT |
| 184431 | Very scant tumor | VCR |
| 184441 | 20 | ENT+VCR |
| 184449 | 20 | ENT+VCR |

**Table S8. Patient history of PDX pleoRMS models**

| **RMS Model** | **Alias** | **Histology** | **Harvest site** | **Mutations** | **Disease stage** | **Time of Biopsy** | **Age** | **Gender** |
| --- | --- | --- | --- | --- | --- | --- | --- | --- |
| CTG-0800 | POS-11074 | pleomorphic | hip | ALK1 [X927X], MYH11, PAX3 [R271C] TP53 [C176F] | IV | first  diagnosis | 38 | M |
| CTG-0329 | POS-10009 | pleomorphic | lung | BRCA1 [L1865R], EP300 [N2379H], RB1 [D210Y], TP53 [N239S] | III | first diagnosis | 47 | M |
| TM01053 | RP2246F | pleomorphic | retroperitoneum | DOT1L [V148L], RET [G691S] | unknown | - | 41 | M |

**Table S9: Statistical summary for CTG-800/POS-1174**

| *day* | *group* | *comparator* | *difference* | *raw CI* | *raw p value* | *tukey CI* | *tukey p value* |
| --- | --- | --- | --- | --- | --- | --- | --- |
| 14 | Control | ENT | 0.38±0.23 | (-0.1, 0.86) | 0.11 | (-0.56, 1.32) | 0.96 |
| 14 | Control | ENT/VCR | 0.64±0.23 | (0.16, 1.12) | 0.01 | (-0.3, 1.58) | 0.41 |
| 14 | Control | VCR | -0.01±0.23 | (-0.49, 0.47) | 0.96 | (-0.95, 0.93) | 1 |
| 14 | ENT | ENT/VCR | 0.26±0.23 | (-0.22, 0.74) | 0.27 | (-0.68, 1.2) | 1 |
| 14 | ENT | VCR | -0.39±0.23 | (-0.87, 0.08) | 0.1 | (-1.34, 0.55) | 0.95 |
| 14 | ENT/VCR | Vincristine | -0.65±0.23 | (-1.13, -0.18) | 0.01 | (-1.6, 0.29) | 0.38 |

**Table S10: Statistical summary for CTG-0329/POS-1009**

| *day* | *group* | *comparator* | *difference* | *raw CI* | *raw p value* | *tukey CI* | *tukey p value* |
| --- | --- | --- | --- | --- | --- | --- | --- |
| 28 | Control | ENT | 0.13±0.11 | (-0.1, 0.35) | 0.26 | (-0.34, 0.59) | 1 |
| 28 | Control | ENT/VCR | 0.07±0.11 | (-0.15, 0.3) | 0.52 | (-0.39, 0.54) | 1 |
| 28 | Control | VCR | 0±0.11 | (-0.22, 0.22) | 1 | (-0.46, 0.46) | 1 |
| 28 | ENT | ENT/VCR | -0.05±0.11 | (-0.28, 0.17) | 0.63 | (-0.52, 0.41) | 1 |
| 28 | ENT | VCR | -0.13±0.11 | (-0.35, 0.1) | 0.26 | (-0.59, 0.34) | 1 |
| 28 | ENT/VCR | VCR | -0.07±0.11 | (-0.3, 0.15) | 0.52 | (-0.54, 0.39) | 1 |

**Table S11: Statistical summary for RP2246F/TM01053**

| *day* | *group* | *comparator* | *difference* | *raw CI* | *raw p value* | *tukey CI* | *tukey p value* |
| --- | --- | --- | --- | --- | --- | --- | --- |
| 27 | Control | ENT | 0.22±0.12 | (-0.02, 0.47) | 0.08 | (-0.28, 0.73) | 1 |
| 27 | Control | ENT/VCR | 0.27±0.12 | (0.02, 0.52) | 0.03 | (-0.23, 0.77) | 0.96 |
| 27 | Control | VCR | 0.21±0.13 | (-0.05, 0.46) | 0.11 | (-0.3, 0.72) | 1 |
| 27 | ENT | ENT/VCR | 0.05±0.12 | (-0.19, 0.28) | 0.7 | (-0.42, 0.51) | 1 |
| 27 | ENT | VCR | -0.02±0.12 | (-0.25, 0.22) | 0.88 | (-0.5, 0.46) | 1 |
| 27 | ENT/VCR | VCR | -0.06±0.12 | (-0.3, 0.17) | 0.59 | (-0.54, 0.41) | 1 |

**Table S12: Histological markers of differentiation in PDX pleoRMS (CTG-800) mice**

| Serial number of pleoRMS tumor (PDX) type | Percentage rhabdomyoblasts | Treatment |
| --- | --- | --- |
| 184871 | Abundant | DMSO |
| 184890 | Abundant | DMSO |
| 184873 | Abundant | ENT |
| 184888 | Abundant | VCR |
| 184889 | Abundant | VCR |
| 184883 | Abundant | ENT+VCR |
| 184885 | Abundant | ENT+VCR |

**Table S13. Primers for RT-PCR**

| sgRNA name | sgRNA matched gDNA sequences (5' to 3') |
| --- | --- |
| Hdac1_e5.1 | TTACGTCAATGACATCGTCC |
| Hdac1_e6.1 | TTACGTCAATGACATCGTCC |
| Hdac1_e7.1 | ATTGATATTCACCATGGCGA |
| Hdac1_e8.1 | TGGCTTAAAGATGGCTTCAT |
| Hdac1_e9.1 | TCAGATTGAAGCAACCTAAC |
| Hdac10_e6.1 | GGTCATCGTTGAAGATATAC |
| Hdac10_e6.2 | GTCGACTGGGATGTCCACCA |
| Hdac10_e7.1 | TCTTGGCACCGCTATGAGCA |
| Hdac10_e8.1 | GATGGGAAATGCCGACTATT |
| Hdac10_e9.1 | TGGATTTGACTCTGCTATCG |
| Hdac11_e3.1 | GCCTCGTGTGCACCACCAGC |
| Hdac11_e4.1 | TGCCTCCAGTCTGGGTCCGC |
| Hdac11_e6.1 | ACTGCTCCAGTGACCGTGGT |
| Hdac11_e7.1 | TTCCTGTTTGAACGCGTGGA |
| Hdac11_e8.1 | CTTTAGCAAAGCGATCCCCA |
| Hdac2_e6.1 | CCGTCTCATTCCATAAATAT |
| Hdac2_e7.1 | TCATCTATACCATCTCTCAT |
| Hdac2_e8.1 | GATGTACCAGCCTAGCGCGG |
| Hdac2_e8.2 | GCAGCACCACCGCGCTAGGC |
| Hdac2_e9.1 | CCATTGCTGATGCTCGGTGG |
| Hdac3_e10.1 | TCCCTGGGCTGTGATCGATT |
| Hdac3_e7.1 | CCACCATGGTGACGGGGTTC |
| Hdac3_e7.2 | TATCAATGTAGAGCACCCGA |
| Hdac3_e8.1 | CTCAATGTGCCCTTACGAGA |
| Rosa | GAAGATGGGCGGGAGTCTTC |
| Rpa3 | ACGGGCCGGTCGATATACTG |

**Supplementary References**

1. Hooper JE, Cantor EL, Ehlen MS, Banerjee A, Malempati S, Stenzel P, Woltjer RL, Gandour-Edwards R, Goodwin NC, Yang Y *et al*: **A Patient-Derived Xenograft Model of Parameningeal Embryonal Rhabdomyosarcoma for Preclinical Studies**. *Sarcoma* 2015, **2015**:826124.
